# Vacuum Packaging Can Extend Fresh Color Characteristics of Beef Steaks during Simulated Display Conditions

**DOI:** 10.3390/foods11040520

**Published:** 2022-02-11

**Authors:** Tristan M. Reyes, Madison P. Wagoner, Virginia E. Zorn, Madison M. Coursen, Barney S. Wilborn, Tom Bonner, Terry D. Brandebourg, Soren P. Rodning, Jason T. Sawyer

**Affiliations:** 1 Department of Animal Sciences, Auburn University, Auburn, AL 36849, USA; tzr0039@auburn.edu (T.M.R.); mpw0035@auburn.edu (M.P.W.); vez0001@auburn.edu (V.E.Z.); madison.coursen@auburn.edu (M.M.C.); wilbobs@auburn.edu (B.S.W.); tdb0006@auburn.edu (T.D.B.); rodnisp@auburn.edu (S.P.R.); 2 Winpak Ltd., 100 Saulteaux Crescent, Winnipeg, MB R3J 3T3, Canada; tom.bonner@winpak.com

**Keywords:** instrumental color, overwrapped packaging, simulated retail display, TBARS, vacuum packaging

## Abstract

Packaging technology is evolving, and the objectives of this study were to evaluate instrumental surface color, expert color evaluation, and lipid oxidation (TBARS) on beef *longissimus lumborum* steaks packaged in vacuum-ready packaging (VRF) or polyvinyl chloride (PVC) overwrap packaging. Paired strip loins (Institutional Meat Purchasing Specifications # 180) were cut into 2.54-cm-thick steaks and assigned randomly to one of two packaging treatments, VRF or PVC. Steaks packaged in VRF were lighter in color (*p* < 0.05) as the display period increased, whereas steaks packaged in PVC became darker (*p* < 0.05). Redness (a*) values were greater (*p* < 0.05) for PVC steaks until day 5, whereas VRF steaks had a greater (*p* < 0.05) surface redness from day 10 to 35 of the display period. Calculated spectral values of red to brown were greater (*p* < 0.05) for steaks in VRF than PVC. In addition, expert color evaluators confirmed VRF steaks were less brown and less discolored (*p* < 0.05) from day 5 to 35 of the display. Nonetheless, lipid oxidation was greater (*p* < 0.05) for PVC steaks from day 10 through day 35 of the display. Results from this study suggest that the use of vacuum packaging for beef steaks is plausible for maintaining surface color characteristics during extended display periods.

## 1. Introduction

Vacuum packaging using form-and-fill technology is a packaging method that is becoming one of the most prominent packaging systems in use within the retail meat industry [1]. Unfortunately, previous research focused on form-and-fill vacuum packaging for use with fresh meat storage in a retail setting is limited. Previous efforts in vacuum packaging uses for fresh meat have focused on using bag or skin technologies [2]. Form-and-fill packaging systems use one film to construct a pouch with time, pressure, and heat. After forming the pouch, meat products are placed into the pouch and a second film is overlayed and sealed within the vacuum chamber. Furthermore, vacuum packaging has accounted for 40% of packaging types within meat cases, with most products packaged using a roll-stock machine [1]. It has been noted that PVC overwrapped packaged beef has decreased in use by 46% from 2018 to 2021 [1]. 

While the meat surface color is still regarded as one of the greatest determining factors consumers utilize when purchasing fresh beef in the retail setting [3,4], packaging technologies are pivotal in maintaining the surface color of fresh meat. PVC is a packaging method used with fresh meat that allows oxygen and other gasses to permeate through the film in large quantities allowing oxygen to bind with myoglobin. The oxymyoglobin state of beef is often correlated with a fresher and more wholesome product by consumers due to a bright cherry red color [5]. Creating a shift from the current industry’s primary packaging methods of PVC to vacuum packaging is unclear; however, many advantages such as the extension of shelf life and color stability may exist with the use of vacuum packaging in fresh meat applications. Vacuum packaging allows meat products to remain more color stable over extended periods of time within retail coolers [6]. Reportedly, vacuum packaging has been known to extend the storage period of fresh meat products by reducing the amount of residual oxygen within the package [7,8].

With the ability to extend fresh meat storage through the use of vacuum packaging, it is a packaging system that is quickly becoming an essential part of the solution for meeting sustainability programs and reducing food waste for the meat industry. Food waste has been characterized as edible food that is not consumed and often discarded by consumers or retailers [9]. It has also been reported that meat, poultry, and fish were the top food groups contributing to an estimated food loss approaching $48 billion in 2010 [9]. In addition, approximately 43 billion pounds of food at the retail level and 90 billion pounds at the consumer level have not been consumed [9]. Aside from food loss and food waste issues that still reside within the meat and food industry, there exist excessive food packaging materials entering the waste management system destined for landfills. In 2017, there were approximately 26.3 billion pounds of beef, 25.6 billion pounds of pork, and 42.2 billion pounds of chicken that American meat companies processed [10]. 

The packaging of fresh meat products is a necessity for the purpose of maintaining a fresh and wholesome product during retail display for consumer purchases. With the volume of packaging necessary to address the meat industry’s demand of packaged meat products, it is essential that a packaging option be investigated for extending storage times of meat products. New packaging technologies could assist in reducing the volume of markdowns and throwaways that occur at the retail counter. A large percentage of fresh meat has been packaged with a form-and-fill roll stock machine, which utilizes multi-layered packaging films [11]. Multi-layered vacuum packaging is constructed with a wide variety of materials that can include amorphous polyethelene terephthalate, polyolefines, ethylene vinyle alcohol, polyvinylidene di-chloride, and nylon [10,11,12,13]. Currently, the ability to recycle multi-layered films lacks economic viability due to the nature of the film layering [14].

Nonetheless, multi-layered vacuum packaging films are growing in popularity for vacuum packaging platforms; unfortunately, these packaging films are often constructed without sustainable or recycle-ready materials. Limitations in recycle-ready packaging materials can create difficulties downstream from the consumer with sustainable meat packaging due to challenges in the delamination process of multi-layered films [10,15]. Nevertheless, an investigation into using multi-layered films is a necessity to extend the fresh-meat shelf life. With a need for greater storage periods of fresh meat by retailers, customers, and consumers, the agriculture industry could focus its efforts on becoming more sustainable through innovative developments of packaging materials for meat and meat products. Therefore, the objectives of the current study were to investigate the feasibility of using VRF vacuum packaging film in place of PVC overwrapping on beef strip loin steaks and the subsequent impacts on surface color characteristics during a simulated retail display period.

## 2. Materials and Methods

### 2.1. Raw Materials

Cattle (*n* = 7) were harvested under simulated commercial conditions according to USDA humane slaughter standards at the Auburn University Lambert Powell Meat Laboratory after a 12 h rest period. After harvest, carcasses were chilled for 48 h at 2 °C. Following carcass chilling, beef carcasses were subsequently fabricated into left- and right-side paired (IMPS # 180) boneless beef strip loins, vacuum packaged (3 mil, Clarity Vacuum Pouches, Kansas City, MO, USA), and stored in the absence of light for 10 days to simulate boxed beef fabrication and logistics. After aging, beef strip loins were cut into 2.54-cm-thick steaks (N = 112 steaks/packaging treatment) using a BIRO bandsaw (Model 3334, BIRO Manufacturing Company, Marblehead, Ohio, USA). At the time of steak cutting, steaks from each loin were allocated randomly to one of two packaging treatments, VRF or PVC. The allocated steaks were placed onto a plastic tray and allowed to bloom for 30 min prior to packaging.

### 2.2. Packaging and Simulated Display Conditions

After steak portioning, steaks allocated to vacuum packaging (VRF) were packaged using a Reiser form-and-fill vacuum packaging machine (Optimus OL0924, Variovac, Zarrentin, Germany) and sealed. Steaks were packaged in VRF packaging films (O_2_ transmission rate = 0.8 cc/sq. m^2^/24 h/atm). Steaks allocated to traditional overwrapping (PVC) were placed onto a foam tray (2s, Genpak, Charlotte, NC, USA) with an absorbent moisture pad (DRI-LOC AC-50, Novipax, Oak Brook, IL, USA) and wrapped by hand with a polyvinyl chloride film (O_2_ transmission rate = 14,000 cc O_2_/m/24 h/atm).

Packaged steaks were placed onto lighted shelves within a refrigerated retail display case (Model TOM- labels 60DXB-N, Turbo Air Inc., Long Beach, CA, USA). Packages of steaks were displayed for 35 days at 3 °C ± 1.2 °C, and the case temperature throughout the display period was monitored with temperature data recorders (Model-TD2F, ThermoWorks, American Fork, UT, USA) placed on the center of each display shelf. Packages of steaks were displayed on shelves under continuous LED lighting with an intensity of 2297 lux for each shelf. Lighting intensity was measured (ILT10C, International Light Technologies, Peabody, MA, USA) throughout the duration of the simulated display period. Additionally, packages of steaks were distributed evenly across all shelves and rotated daily from side to side and front to back to simulate consumer movement. Fresh meat characteristics of instrumental color, surface color, lipid oxidation, purge loss, and pH were measured on days 0, 5, 10, 15, 20, 25, 30, and 35 throughout the simulated display period.

### 2.3. Instrumental Color

Throughout the 35-day simulated retail display period, the instrumental surface color was measured on packaged steaks (*n* = 28) with a HunterLab MiniScan EZ colorimeter, Model 45/0 LAV (Hunter Associates Laboratory Inc., Reston, WV, USA). Prior to surface color readings, the colorimeter was standardized using a black and white tile. Instrumental color values were determined from the mean of three readings through the surface of each unopened package using illuminant A, an aperture of 31.8 mm, and a 10° observer. Packages of steaks were evaluated for lightness (L*), redness (a*), and yellowness (b*) using the Commission Internationale de l’ Eclairage guidelines for surface color [16]. In addition, the hue angle was calculated as tan^−1^ (b*/a*), with a greater value indicative of the surface color shifting from red to yellow. Chroma (C*) was calculated as √a*^2^ + b*^2^, where a larger value indicates a more vivid color. Lastly, reflectance values within the spectral range of 400 to 700 nm were used to capture the surface color changes from red to brown by calculating the reflectance ratio of 630 nm:580 nm and the relative values of deoxymyoglobin (DMb = {[1.395 − ({A572 − A700}/{A525 − A700})]} × 100), metmyoglobin (MMb = {2.375 × [1 − ({A473 − A700}/{A525 − A700})]} × 100), and oxymyoglobin (OMb = DMb − MMb) according to color guidelines previously described [17]. 

### 2.4. Expert Color Evaluation 

A five-member, expert color panel was used to evaluate the surface color of packaged beef boneless strip steaks during the simulated retail display period. Color measuring experts used anchors for scoring surface color discoloration previously described and modified from meat color guidelines [12]. At 16:00 h on the day of simulated display, experts rated surface color changes for steaks (*n* = 28) every 5 days for 35 days of refrigerated storage. Surface color ratings were created for steaks packaged under vacuum (VRF) for the initial beef color (1 = extremely bright purple-red, 2 = bright purple-red, 3 = moderately bright purple-red, 4 = slightly purple-red, 5 = slightly dark purple, 6 = moderately dark purple, 7 = dark purple, 8 = extremely dark purple), whereas packages of PVC overwrapped steaks were rated for the initial beef color (1 = extremely bright cherry-red, 2 = bright cherry-red, 3 = moderately bright, 4 = slightly bright cherry-red, 5 = slightly dark cherry-red, 6 = moderately dark red, 7 = dark red, 8 = extremely dark red). Both VRF- and PVC-packaged steaks were rated for the amount of browning (1 = no evidence of browning, 2 = dull, 3 = grayish, 4 = brownish gray, 5 = brown, and 6 = dark brown) and percent (%) discoloration (1 = no discoloration [0%], 2 = slight discoloration [1–20%], 3 = small discoloration [21–40%], 4 = modest discoloration [41–60%], 5 = moderate discoloration [61–80%], 6 = extensive discoloration [81–100%]). 

### 2.5. Purge Loss and Fresh Muscle pH 

Prior to conducting lipid oxidation analysis, steaks were removed from their respective packaging materials, blotted dry, and weighed on an analytic balance (PB3002-S, Mettler Toledo, Columbus, OH, USA). Purge loss was calculated as [(packaged steak weight − steak weight) ÷ packaged steak weight × 100)]. After capturing the purge loss for each steak, fresh muscle pH was measured in duplicate with a glass electrode inserted into two random locations within the steak and attached to a pH meter (Model-HI99163, Hanna Instruments, Woonsocket, RI, USA). Prior to measuring, the pH probe was calibrated (pH 4.0 and 7.0) using 2-point standard buffers (Thermo Fisher Scientific, Chelmsford, MA, USA) and again after 10 readings. 

### 2.6. Lipid Oxidation 

Packaged steaks (*n* = 56) were removed from their packaging material and sampled for 2-thiobarbituric acid reactive substances (TBARS) using a previously described method [18]. Steaks were trimmed of all external fat and connective tissue then minced together to form a uniform sample of the entire steak. Approximately 2 g of minced muscle was homogenized with 8 mL of cold (1 °C) 50 mM phosphate buffer (pH of 7.0 at 4 °C) containing 0.1% EDTA, 0.1% n-propyl gallate, and 2 mL trichloroacetic acid (Sigma-Aldrich, Saint Louis, MO, USA). Homogenized samples were subsequently filtered through Whatmann No. 4 filter paper, and duplicate 2-mL aliquots of the clear filtrate were transferred into 10-mL borosilicate tubes, mixed with 2 mL of 0.02 M 2-thiobarbituric acid reagent (Sigma-Aldrich, Saint Louis, MO, USA) then boiled for 20 min. After boiling, tubes were placed into an ice bath for 15 min. Absorbance was measured at 533 nm with a spectrophotometer (Turner Model–SM110245, Barnstead International, Dubuque, IA, USA) and multiplied using a factor of 12.21 to obtain the TBARS value (mg malonaldehyde/kg of meat). 

### 2.7. Statistical Analysis

Data were analyzed with the GLIMMIX procedures of SAS (ver. 9.4; SAS Institute Inc. Cary, NC, USA) with treatment serving as the lone fixed effect and replication serving as the random effect for instrumental color, expert color, lipid oxidation, purge loss, and pH. All data were analyzed in a modified randomized design with steak serving as the experimental unit. For expert surface color rating data, the expert color panelist was included as a random factor, and panelist × day of display was included as a random, repeated factor (with a first-order autoregressive covariance structure). Least-squares means were generated, and when significant (*p* ≤ 0.05) F-values were observed, least-squares means were separated using a pair-wise *t*-test (PDIFF option).

## 3. Results and Discussion

### 3.1. Instrumental Beef Color

The instrumental surface color of packaged steaks was measured throughout a 35-day simulated retail period. An interaction of the packaging method × day of display on steak surface lightness (L*) occurred (Table 1). Steaks packaged in PVC were lightest (*p* < 0.05) on day 0 and became darker as the length of display period increased (Table 1). However, from day 20 through day 35 of the display, steaks packaged using VRF were lighter (*p* < 0.05) than steaks packaged using PVC methods (Table 1). Additionally, surface redness (a*) for beef steaks packaged in PVC were redder (*p* < 0.05) from day 0 through day 15 of the display period (Table 1), whereas steaks packaged in VRF became significantly redder (*p* < 0.05) until the conclusion of the study on day 35 (Table 1). Greater a* values are indicative of a redder fresher color and have a greater consumer appeal at the time of the consumers’ purchasing decision. PVC-packaged steaks maintained greater (*p* < 0.05) values for yellowness (b*) throughout the duration of simulated retail display than steaks packaged in VRF (Table 1). The changes in surface color for steaks packaged using VRF indicated surface lightness and redness were more stable throughout the entire simulated retail period than steaks packaged in PVC. As expected during a simulated retail period, fresh steaks packaged in an oxygen-rich permeable method such as PVC will have a brighter surface color initially. Similar findings have reported that ground beef packaged using PVC methods resulted in greater L* values on day 0, along with greater a* and b* through only 50% of the display period [5] when displayed up to 35 days. Moreover, ground beef patties when packaged with PVC materials have recorded similar results, indicating a* values will decline within the first 5 days of the display period [19]. However, a* values for ground beef patties packaged using a vacuum packaging platform have been reported to increase throughout a display period [19]. Furthermore, a study evaluating the surface color of beef steaks indicated a* values were greater for vacuum packaging rather than other packaging types at the conclusion of a 35-day study [20]. It appears the results for b* values of ground beef and steaks are consistent with the current study, resulting in a decline during a 5-day retail storage period when packaged in PVC. Regardless of the fluctuation of yellowness, the current and previous results suggest b* values are less stable regardless of the packaging method [5,19,20,21]. 

There was a packaging method × day of display interaction for surface color chroma (C*) and hue angles (Table 1). The instrumental surface color of steaks packaged in PVC was more vivid (*p* < 0.05) on day 0 but C* values declined as the duration of display increased. However, steaks packaged with VRF became more vivid (*p* < 0.05) from day 25 through 35 of the simulated retail display period (Table 1). In addition, steaks packaged with PVC had greater (*p* < 0.05) hue angles indicative of a surface color shift from red to yellow from day 5 through 35. It appears that the reduction in oxygen exposure for steaks in VRF packages protected the surface color of steaks by sustaining the vividness and reducing the shift from red to yellow. Similar results for fresh packaged beef C* and hue angle values have been reported to decline during the initial 10 days of a simulated display period when using an oxygen-rich packaging method such as PVC [22]. Changes in surface color values for the hue angle and C* can be used as a great indicator for observing meat discoloration in retail display settings [19,20,21,22,23,24]. Interestingly, C* (vividness) for steaks packaged in VFR in the current study differ from previous C* results that did not differ throughout a 35-day display period [23]. It should be noted that as the percentage of oxygen exposure to the steak surface increases a reduction in the hue angle and C* will likely occur during retail display periods as the surface color shifts from red to brown with the formation of metmyoglobin [22,23,24]. 

The interactive influence for packaging method × day of display remained for calculated spectral values of red to brown (630:580 nm) and relative forms of myoglobin (Table 1). Red to brown values were greater (*p* < 0.05) for steaks packaged in PVC until day 5 of the simulated display period. However, from day 10 through 35, PVC-packaged steaks’ surface color showed a greater shift from red to brown. Steaks packaged in VRF had less (*p* < 0.05) discoloration from red to brown after day 5 through day 35 (Table 1). Previous studies have [25] reported similar findings indicating a decline in calculated red to brown values throughout 7 days of simulated display for beef packaged in PVC [25]. It is expected that calculated spectral values for the surface color of fresh beef will shift from a brighter red to brown as the duration of a simulated retail display increases. Steaks packaged in VRF had the greatest (*p* < 0.05) amount of calculated metmyoglobin (MMb) on day 0 (Table 1). However, as expected from days 5 to 35, steaks packaged using PVC had greater (*p* < 0.05) calculated relative values for MMb. As expected, steaks packaged in VRF had greater (*p* < 0.05) calculated deoxymyoglobin (DMb) values throughout the entire simulated retail display period (Table 1) because of limited oxygen exposure. Interestingly, calculated relative values of oxymyoglobin (OMb) were greater (*p* < 0.05) for steaks packaged using PVC packaging materials throughout the entire simulated retail display period (Table 1). The results for calculated spectral values reported are likely due to the oxygen permeability of the PVC package resulting in greater exposure of the steak surface to an oxygen-rich atmosphere. Greater formations of MMb in PVC have been associated with greater amounts of lipid oxidation [26,27] and the relationship of oxidation during the transition of myoglobin pigment from OMb to MMb [26,27,28].

### 3.2. Expert Color Evaluation

Fresh steaks were evaluated by experts for visual surface color variations during a simulated retail display for up to 35 days. However, the evaluation of steaks packaged in aerobic PVC packaging materials was discontinued after day 20 due to total surface color deterioration. An interaction of the packaging method × day of display occurred for the surface color evaluation (Table 2). Trained expert evaluators noted that values for the initial beef color, amount of browning, and surface discoloration deteriorated (*p* < 0.05) for steaks packaged in PVC from day 5 through day 20 (Table 2). The surface color of steaks packaged in PVC materials became darker, with a greater amount of browning, and a greater percentage of discoloration as the duration of display increased. As a result of significant surface discoloration, PVC-packaged steaks used for expert color evaluation were discarded on day 20 of the display period. The changes in visual surface color are influential in driving consumer purchasing intent and the lack of storage for PVC steaks may contribute to greater throwaway by the retailer. Steaks packaged in VRF had initial beef colors that decreased (*p* < 0.05), and the amount of browning and surface discoloration were less (*p* < 0.05) than steaks packaged in PVC throughout the duration of the study (Table 2). Interestingly, steaks packaged in VRF were darker at day 0, but the visual steak color turned brighter purple red with less browning and surface discoloration throughout a 35-day simulated retail period. Results from the current study agree with previous findings when using vacuum packaging. Beef’s surface color tends to remain visually stable throughout the duration of the study, whereas high-oxygen packaging of fresh beef can show rapid color deterioration [29]. The color stability of fresh beef is dependent on controlling countless factors such as pH, temperature, light, lipid oxidation, residual oxygen, MMb-reducing systems, reducing equivalents, and the oxygen consumption rate [30,31]. It is plausible that the transformation from OMb to MMb in PVC-packaged steaks was due to greater amounts of lipid oxidation. Furthermore, limited surface color variation of steaks packaged in VRF may be attributed to a lack of residual oxygen within the packaging, influencing and reducing the amount of oxidation occurring in vacuum-packaged fresh beef products. 

### 3.3. Lipid Oxidation

**Figure 1:**
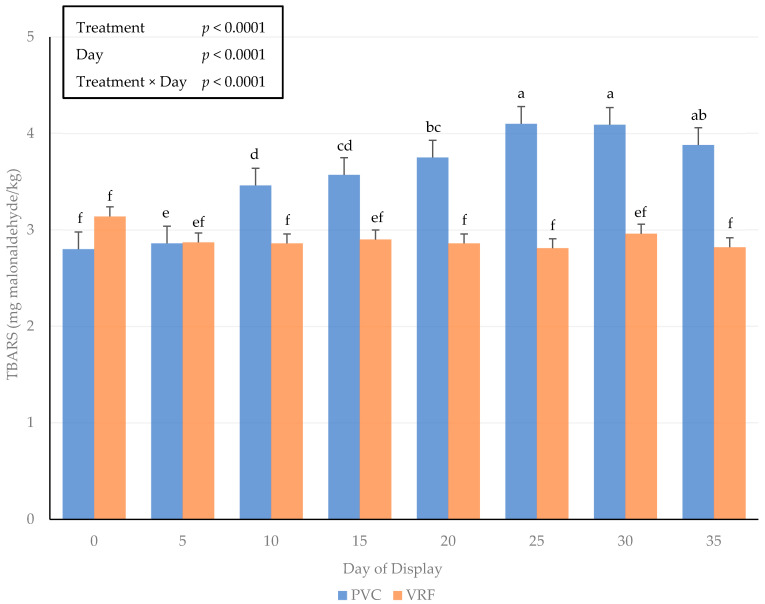
Interactive influence of packaging method × day of display for 2-Thiobarbituric acid reactive substances (TBARS) on beef strip loin steaks during a simulated retail display. Bars lacking common letters differ (*p* ≤ 0.05).

There was an interactive effect of the packaging method × day of display for lipid oxidation on fresh beef steaks (Figure 1). The packaging method did not alter (*p* > 0.05) lipid oxidation through day 5 of the simulated retail display period. However, from days 10 through 35 of the storage period, lipid oxidation was greater (*p* < 0.05) for steaks packaged using PVC methods. Lipid oxidation of fresh steaks using PVC packaging from the current study agrees with previous simulated retail storage studies measuring an expected storage period in a retail setting of 3 to 7 days [32]. The exposure to greater amounts of oxygen across the packaging material can result in increased catalysis of lipid oxidation [33,34]. Moreover, greater lipid oxidation can be correlated to reduced consumer palatability due to the deterioration of the surface color and accumulation of off flavors [35]. Unfortunately, the evaluation of sensory taste characteristics was not completed during the current study, but future studies on the extended storage of fresh beef influencing lipid oxidation and sensory characteristics would be warranted. 

### 3.4. Purge Loss

**Figure 2:**
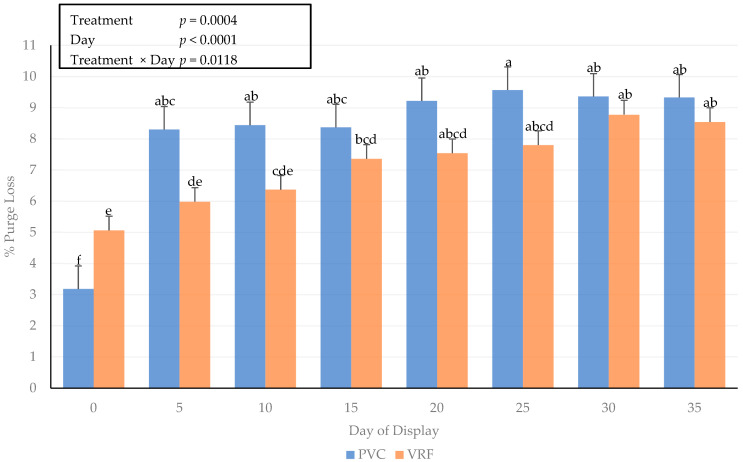
Interactive influence of packaging method × day of display for purge loss (%) on beef strip loin steaks during a simulated retail display. Bars lacking common letters differ (*p* ≤ 0.05).

A packaging method × day of display interaction occurred for the purge loss of fresh beef steaks (Figure 2). The purge loss was greatest (*p* < 0.05) for steaks packaged in PVC materials on day 25 of the simulated display period and the lowest on day 0. The packaging method influenced the purge loss on day 0, with steaks packaged in VRF having a greater (*p* < 0.05) percentage of moisture loss. It is plausible that the method of vacuum packaging using the form-and-fill machine caused more moisture to be pressed out of the steak at the time of package sealing. However, the purge loss in vacuum-packaged meat products can result in an unappealing visual appearance for consumers due to the accumulation of purge in the packaging [36,37]. The results from the current study differ from previous results where values for purge loss using vacuum-packaging platforms were greater than PVC or alternative packaging such as modified atmosphere packaging platforms [38]. 

### 3.5. pH

**Figure 3:**
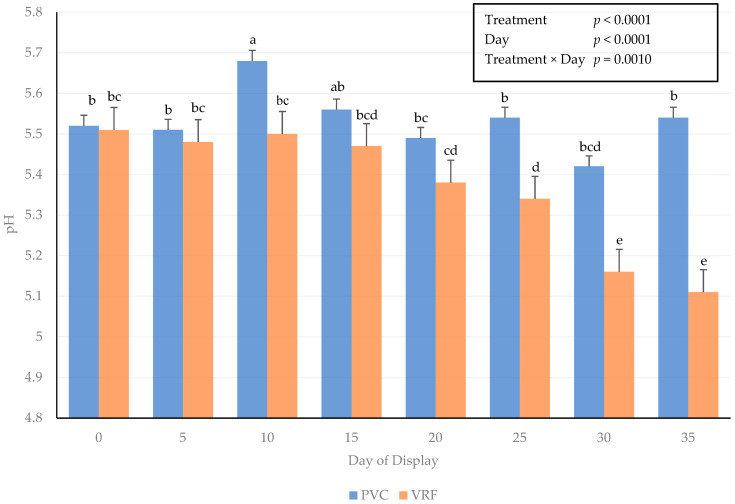
The interactive influence of packaging method × day of display for fresh muscle pH on beef strip loin steaks. Bars lacking common letters differ (*p* ≤ 0.05).

The interactive influence of the packaging method × day of display for fresh muscle pH values is presented in Figure 3. Fresh muscle pH values were recorded within muscle pH values (5.1 to 5.8) throughout the duration of the simulated display period. Values for fresh muscle pH were greatest (*p* < 0.05) on day 10 in steaks packaged using PVC methods. At the time of harvest and before chilling, carcasses were rinsed with an FDA-GRAS (U.S. Food and Drug Administration-Generally Recognized as Safe) organic acid (lactic acid). The combination of vacuum packaging and the organic carcass wash may have contributed to the decline in fresh muscle pH of VRF-packaged steaks, causing a shift in the visual and instrumental surface color variations reported within the current study. Furthermore, it is plausible that pH values for VRF declined due to an increase in lactic acid bacteria that can be present in vacuum-packaged fresh meats. With limited residual oxygen within the vacuum package, favorable conditions for anerobic lactic acid bacteria may have caused fresh muscle pH to decline as lactic acid bacteria populations increased [5,39]. In addition, lactic acid bacteria can be associated with low-pH (<5.8) vacuum-packaged meats due to a lower residual oxygen environment [40]. 

## 4. Conclusions

It is feasible that the storage of beef strip loin steaks using vacuum packaging, VRF, can provide a longer, fresh, refrigerated storage period than steaks packaged in traditional PVC packaging. It is evident that VRF displayed a more color-stable product throughout the duration of simulated retail display. Additionally, VRF maintained less oxidation throughout the display period, whereas steaks packaged in PVC tended to have greater oxidation leading to greater amounts of surface discoloration in beef products. The current results suggest that the vacuum-packaged film used within the current study is an acceptable replacement to traditional packaging methods of PVC for packaging whole-muscle beef steaks for up to 35 days of refrigerated retail storage. However, additional research should be considered to evaluate the sensory taste profiles of vacuum packaging used for extended storage periods and the implications for flavor characteristics of beef steaks. 

## Figures and Tables

**Table 1:** The interactive impact of packaging method × day of display for instrumental surface color values on fresh beef strip loin steaks during a simulated retail display.

	Day								
	0	5	10	15	20	25	30	35	SEM *
**PVC**									
L* ^1^	46.85 ^a^	45.42 ^abc^	44.26 ^cde^	44.37 ^bcde^	43.31 ^de^	43.08 ^def^	42.47 ^efg^	40.63 ^g^	0.713
a* ^1^	29.57 ^a^	25.40 ^b^	19.33 ^d^	15.93 ^e^	15.69 ^e^	15.79 ^e^	15.93 ^e^	15.99 ^e^	0.704
b* ^1^	21.33 ^a^	19.97 ^b^	17.71 ^c^	15.13 ^d^	14.29 ^de^	14.03 ^de^	13.69 ^e^	13.44 ^e^	0.392
C* ^2^	36.47 ^a^	32.32 ^b^	26.31 ^c^	22.07 ^efg^	21.41 ^g^	21.31 ^g^	21.16 ^g^	21.01 ^gh^	0.672
Hue (°) ^3^	35.76 ^d^	38.22 ^cd^	43.24 ^ab^	43.81 ^a^	42.87 ^ab^	42.16 ^ab^	41.08 ^abc^	40.20 ^bc^	1.185
RTB ^4^	5.28 ^a^	3.94 ^b^	2.57 ^de^	1.92 ^f^	1.99 ^f^	2.03 ^f^	2.07 ^f^	2.22 ^ef^	0.143
MMb ^5^	20.40 ^ef^	28.08 ^d^	40.18 ^abc^	43.22 ^a^	41.09 ^ab^	38.81 ^abc^	37.14 ^bc^	34.59 ^c^	2.081
DMb ^5^	4.89 ^f^	7.68 ^ef^	14.27 ^e^	24.25 ^d^	33.14 ^c^	34.91 ^c^	35.74 ^c^	38.74 ^c^	3.161
OMb ^5^	74.71 ^a^	64.25 ^b^	45.55 ^c^	32.53 ^d^	25.77 ^e^	26.28 ^e^	27.12 ^e^	26.67 ^e^	1.815
**VRF**									
L* ^1^	41.17 ^fg^	42.56 ^efg^	43.32 ^de^	44.86 ^abcd^	45.41 ^abc^	46.37 ^ab^	46.58 ^a^	45.84 ^abc^	0.713
a* ^1^	15.72 ^e^	19.54 ^d^	19.56 ^d^	19.89 ^cd^	20.26 ^cd^	20.55 ^cd^	20.91 ^cd^	21.59 ^c^	0.704
b* ^1^	11.03 ^fg^	9.69 ^h^	9.85 ^h^	10.26 ^gh^	10.48 ^gh^	11.07 ^fg^	11.59 ^f^	12.12 ^f^	0.392
C* ^2^	19.26 ^h^	21.82 ^fg^	21.91 ^fg^	22.39 ^efg^	22.82 ^efg^	23.36 ^def^	23.92 ^de^	24.77 ^cd^	0.672
Hue (°) ^3^	35.13 ^d^	26.41 ^e^	26.71 ^e^	27.28 ^e^	27.31 ^e^	28.26 ^e^	29.00 ^e^	29.32 ^e^	1.185
RTB ^4^	2.12 ^f^	3.18 ^c^	3.10 ^c^	2.90 ^cd^	2.85 ^cd^	2.70 ^d^	2.57 ^de^	2.69 ^d^	0.143
MMb ^5^	34.97 ^c^	14.54 ^g^	15.20 ^fg^	17.20 ^fg^	18.62 ^efg^	20.62 ^ef^	23.56 ^de^	24.18 ^de^	2.081
DMb ^5^	53.28 ^b^	86.97 ^a^	87.40 ^a^	88.35 ^a^	88.95 ^a^	86.65 ^a^	82.89 ^a^	83.98 ^a^	3.161
OMb ^5^	11.75 ^f^	2.69 ^h^	2.90 ^h^	5.67 ^gh^	7.57 ^fgh^	7.26 ^fgh^	6.63 ^gh^	8.16 ^fg^	1.815

^1^ L* Values are a measure of darkness to lightness (larger value indicates a lighter color); a* values are a measure of redness (larger value indicates a redder color); and b* values are a measure of yellowness (larger value indicates a more yellow color). ^2^ C* (Chroma) is a measure of total color (larger number indicates a more vivid color). ^3^ Hue (°) angle represents the change from the true red axis (larger number indicates a greater shift from red to yellow). ^4^ RTB calculated as 630 nm ÷ 580 nm, which represents a change in the color of red to brown (larger value indicates a redder color). ^5^ Calculated percentages of deoxymyoglobin (DMb), metmyoglobin (MMb), and oxymyoglobin (OMb) using relative spectral values. ^a—h^ Mean values within a row and a packaging method lacking common superscripts differ (*p* ≤ 0.05). * SEM, Standard error of the mean. Bold font, the packaging methods investigated.

**Table 2:** Interactive influence of packaging method × day of display for expert surface color evaluation on fresh beef strip loin steaks during a simulated retail display.

	Day of Simulated Display								
	0	5	10	15	20	25	30	35	SEM *
**PVC ^1^**									
Initial Beef Color	1.20 ^h^	3.34 ^f^	4.48 ^e^	5.09 ^c^	6.50 ^b^	--	--	--	0.154
Amount of Browning	1.00 ^e^	1.79 ^c^	3.94 ^b^	3.75 ^b^	4.52 ^a^	--	--	--	0.080
Surface Discoloration	1.09 ^efg^	1.79 ^d^	3.12 ^c^	4.20 ^b^	4.76 ^a^	--	--	--	0.073
**VRF ^2^**									
Initial Beef Color	7.34 ^a^	5.57 ^c^	4.17 ^e^	4.15 ^e^	3.41 ^f^	3.42 ^f^	3.52 ^f^	2.79 ^g^	0.154
Amount of Browning	1.00 ^e^	1.17 ^de^	1.30 ^d^	1.00 ^e^	1.23 ^d^	1.00 ^e^	1.00 ^e^	1.20 ^de^	0.080
Surface Discoloration	1.03 ^fg^	1.21 ^ef^	1.01 ^g^	1.01 ^g^	1.23 ^e^	1.00 ^g^	1.00 ^g^	1.06 ^efg^	0.073

^1^ PVC color anchors: Initial Beef Color (1 = Extremely bright cherry-red to 8 = Extremely dark red); Amount of Browning (1 = No Evidence of Browning to 6 = Dark Brown); Surface Discoloration (1 = No discoloration (0%) to 6 = Extensive discoloration (81–100%). ^2^ VRF color anchors: Initial Beef Color (1 = Extremely bright purple red to 8 = extremely dark purple red); Amount of Browning (1 = No Evidence of Browning to 6 = Dark Brown); Surface Discoloration (1 = No discoloration (0%) to 6 = Extensive discoloration (81–100%). ^a–h^ Mean values within a row and packaging method lacking common superscripts differ (*p* ≤ 0.05). * SEM, Standard error of the mean. Bold font, the packaging methods investigated.

## Data Availability

Not Applicable.
